# Artificial intelligence for the general cardiologist

**DOI:** 10.1007/s12471-019-01327-7

**Published:** 2019-08-21

**Authors:** J. Verjans, T. Leiner

**Affiliations:** 1 Royal Adelaide Hospital, Adelaide, SA Australia; 2 South Australian Health and Medical Research Institute, Adelaide, SA Australia; 3 Australian Institute for Machine Learning, University of Adelaide, Adelaide, SA Australia; 4 Department of Radiology, University Medical Centre Utrecht, Utrecht University, Utrecht, The Netherlands

The majority of experts and opinion leaders believe that artificial intelligence (AI) is going to revolutionise many industries, including healthcare [1]. In the short term, the power and potential of AI appear most suitable for complementing human expertise. In other words, machines will help humans do a better job. Consequently, it is anticipated that AI will help with repetitive tasks, in-depth quantification and classification of findings, improved patient and disease phenotyping and, ultimately, with better outcomes for patients, physicians, hospital administrators, insurance companies and governments [2].

**Fig. 1:**
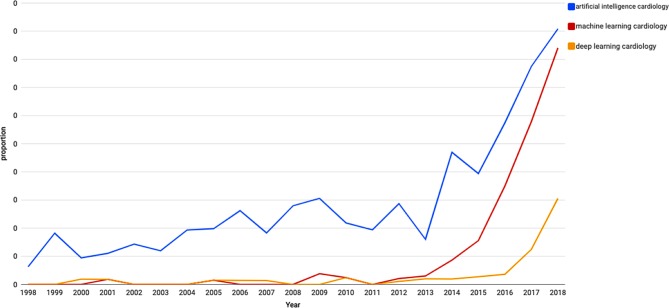
Proportion of citations on PubMed (1998–2018) for each search per year for key terms (source: http://esoerr.github.io/pubmed-by-year)

Despite these promises, the impact of AI in current clinical practice is still limited. However, this could change in the coming years, as illustrated by the significant increase in papers in AI, machine learning and deep learning in cardiology (Fig. 1). Moreover, multiple applications have gained Federal Drug Administration approval in recent years with significant financial support; these are directly related to daily cardiology practice, including automated interpretation of electrocardiograms, automated segmentation and diagnosis (Tab. 1). Common practice in invasive cardiology will be seriously influenced by AI, for example by predicting the outcome of interventions such as transcatheter aortic valve implantation or AI-based non-invasive estimation of the haemodynamic significance (CT fractional flow reserve) of coronary artery stenosis on CT angiography that is being developed by different companies [3–5].

**Table 1:** Federal Drug Administration approvals of artificial intelligence (*AI*) applications relevant for cardiology practice

Year	Company	AI application
2014	Alivecor	Detection of atrial fibrillation
2016	Lumify	Ultrasound image diagnosis
2017	Arterys	Cardiac MRI ventricle segmentation
2017	Bioflux	Detecting arrhythmias
2017	Cardiologs	ECG analysis
2018	Lepu Medical	ECG analysis
2018	Zebra Medical Vision	Automated coronary artery calcification
2018	Physiq Heart Rhythm Module	Detection of atrial fibrillation
2018	Apple	Detection of atrial fibrillation
2018	Bay Labs	Echocardiogram analysis
2019	Verily	ECG feature on Study Watch
2019	Alivecor	Six-lead smartphone ECG
2019	Zebra Medical Vision	Radiographic diagnosis
2019	Aidoc	Flagging pulmonary embolism

This focus issue of the *Netherlands Heart Journal* aims to help general cardiologists explore the state of the art of AI in cardiology. It also aims to increase awareness that it is likely that AI is going to influence and even disrupt daily clinical practice and healthcare in general.

The first part of the issue will focus on past, present and future evidence and gives an overview in the form of two reviews on the impact of AI in cardiology and an area of more immediate impact, cardiovascular imaging [6, 7]. In the second part of this issue, several Dutch flagship AI projects are discussed and demonstrate the potential in various areas of AI as discussed above. These efforts not only concern clinical practical problems such as cardiovascular imaging and risk assessment, but also the need for multidisciplinary collaboration [8] and dedicated data platforms to access and analyse the collected data [9, 10].

According to scientists from every decade since the 1960s, human-like AI should have been achieved within 10–20 years. However, mainly due to a lack of computational power, there have been two so-called ‘winters’ for AI around 1980 and 1993, both after a period of increased interest. But with the recent increase in computational power, smarter use of hardware and data, and new strategies such as deep learning, this appears to have changed for good. Artificial intelligence is likely to make its potential come true in the era of complex data, serving as a conduit to insight for doctors in their patient’s data, improving efficiency and reducing errors.

Let’s plan some randomised clinical trials!
